# Bioinformatic Analysis of Key Genes and Pathways Related to Keloids

**DOI:** 10.1155/2021/5897907

**Published:** 2021-03-23

**Authors:** Siwei Bi, Ruiqi Liu, Beiyi Wu, Linfeng He, Jun Gu

**Affiliations:** ^1^West China School of Medicine, Sichuan University, Chengdu, Sichuan 610041, China; ^2^Department of Burn and Plastic Surgery, West China Hospital, Sichuan University, Chengdu, Sichuan 610041, China; ^3^Department of Cardiovascular Surgery, West China Hospital, Sichuan University, Chengdu, Sichuan 610041, China

## Abstract

**Background:**

The pathophysiology of keloids is complex, and the treatment for keloids is still an unmet medical need. Our study is aimed at identifying the hub genes among the differentially expressed genes (DEGs) between normal skin tissue and keloids and key pathways in the development of keloids.

**Materials and Methods:**

We downloaded the GSE92566 and GSE90051 microarray data, which contain normal skin tissue and keloid gene expression data. GSE92566 was treated as a discovery dataset for summarizing the significantly DEGs, and GSE90051 served as a validation dataset. Gene Ontology, Kyoto Encyclopedia of Genes and Genomes pathway, Reactome enrichment analysis, gene set enrichment analysis, and gene set variation analysis were performed for the key functions and pathways enriched in DEGs. Moreover, we also validated the hub genes identified from the protein-protein interaction network and predicted miRNA-hub gene interactions.

**Results:**

117 downregulated DEGs and 204 upregulated DEGs in GSE92566 were identified. Extracellular and collagen-related pathways were prominent in upregulated DEGs, while the keratinization-related pathway was associated with downregulated DEGs. The hub genes included COL5A1, COL5A2, and SERPINH1, which were also validated in GSE90051.

**Conclusion:**

This study identified several hub genes and provided insights for the underlying pathways and miRNA-hub gene interactions for keloid development through bioinformatic analysis of two microarray datasets. Additionally, our results would support the development of future therapeutic strategies.

## 1. Introduction

Keloid is one of the fibroproliferative disorders as a result of deep injuries that reach the reticular dermis, such as trauma, burn, surgery, chickenpox, vaccination, and insect bite [1]. A typical manifestation of keloid could be keloidal collagen or collagen bundle accumulation accompanied by pain, pruritus, hyperesthesia, limitation of joint movement, and/or cosmic problems [2]. Therapies including surgery, radiotherapy, antimetabolic agents, and compression therapy are used as treatments for keloid. However, it is still challenging to cure keloid because of its high recurrence rate [3].

Many researchers have investigated the pathophysiology of keloids from different standpoints, including cytokines, hypoxia, and genetics [4]. However, no definitive conclusion has been derived yet. It is believed that genetics plays a significant part in keloid formation, due to the varying occurrence rate of keloid between ethnicities and families [4]. The incidence of keloid ranges between 0.15% in east Asians and 6~16% in Africans [5, 6]. Besides, family members of keloid patients have a greater possibility to suffer keloids than individuals with a negative family history. The severity of keloids and the number of anatomical sites where keloids appear are also linked to family history [7]. In spite of a seemingly obvious genetic predisposition for this disease, genes related to keloid pathogenesis are still unknown. Some chromosomal regions were found to possibly contain genes related to family keloids [8], including 2q23 in Japanese and 7p11 in African American populations [9]. Some human leukocyte antigen (HLA) genes have also been demonstrated to correlate with keloid formation [10]. Other studies have attributed the development of keloids to genetic polymorphisms and mutations [9, 10].

In recent years, there has been an increase in the number of publicly available transcriptome data with respect to keloids; some studies have examined the transcriptome data in keloids and adjacent nonlesional (NL) skin in African Americans and Japanese, respectively [11, 12]. In this study, our objective was to identify the differentially expressed genes (DEGs) correlated with keloids and the pathways they participate in, which would give vital insights into the potential pathogenesis in keloids. Meanwhile, the hub genes and their interaction with miRNAs would be demonstrated for a more comprehensive understanding of the potential regulatory network in keloids.

## 2. Materials and Methods

### 2.1. Microarray Data Download and Preprocessing

We downloaded GSE92566 and GSE90051 microarray data from NCBI GEO (http://www.ncbi.nlm.nih.gov/geo/). GSE92566 contains gene expression data for 7 African American samples: 1 newly formed keloid, 3 keloid lesions, and 3 adjacent nonlesion samples using Affymetrix Human Genome U133 Plus 2.0 Array. GSE90051 documented transcriptional profiling from 7 Japanese patients with keloids by Agilent-014850 Whole Human Genome Microarray 4x44K G4112F. Raw data of both datasets were preprocessed using R software. Specifically, we implemented the robust multiarray average (RMA) algorithm in oligo package [13] and preprocessing pipeline in Limma package [14] for GSE92566 and GSE90051, respectively, based on their platforms. The annotation for the probes and clinical trait information were downloaded using the GEOquery package [15]. GSE92566 and GSE90051 were treated as discovery and validation datasets, respectively, since the platform GSE90051 used only gives the normalized log10 ratio between test and reference while GSE92566 contains the expression value of each probe.

### 2.2. Identification of DEGs

Firstly, we excluded the newly formed keloid sample in GSE92566 to achieve better consistency in the data. We used the Limma package to identify the differentially expressed probes in the keloids compared with the normal tissue samples [16]. The authors chose the largest value of log2 fold change for each unique probe name and thus for each gene. The Benjamini-Hochberg (BH) method was used to execute the multiple testing correction for accessing the adjusted *p* value [17]. Absolute log2 fold changes greater than 1.5 and adjusted *p* value less than 0.05 were selected as the threshold values and separated the upregulated genes and downregulated genes. The significant DEGs were illustrated using R package “pheatmap” [18]. A list of DEGs was documented for the following analysis.

### 2.3. Gene Ontology (GO), Kyoto Encyclopedia of Genes and Genomes (KEGG) Pathway, and Reactome Enrichment Analysis

GO analysis has been a popular method to elucidate potential biological processes (BP), molecular functions (MF), and cellular components (CC) associated with the genes. The KEGG pathway database contains information about the mechanism of networking between molecules or genes. It complements the majority of the current molecular biology databases with further information, including information on individual genes [19]. Biological pathways enriched in the target genes were also interrogated with the Reactome pathway database. We performed the enrichment analysis in Metascape (http://metascape.org/), a gene annotation and analysis resource [20]. The threshold for *p* value was set at 0.01, and the minimum enrichment score was 1.5.

### 2.4. Gene Set Enrichment Analysis (GSEA) and Gene Set Variation Analysis (GSVA) on the Selected Modules

GSEA [21] was used to associate the potential gene signature sets comparing the keloid versus skin tissue. The expression profile of GSE92566 was ranked in order of log fold change, and GSEA was performed using the R package XGR [22] with the KEGG databases and 20,000 permutations. In addition, we used GSVA implemented in the R package GSVA [23] to explore differences between the keloid and normal skin in biological pathways. GSVA defines a set of genes based on a list of biological pathways or function terms. We chose the “h.all.v7.0.symbols.gmt” downloaded from “http://software.broadinstitute.org/gsea/index.jsp.” Each gene set's value represents the up- or downregulation of the specific term in that sample. Systematic expression change of genes in the same terms or similar processes indicates the variation in the activity of a specific pathway as a whole.

### 2.5. Protein-Protein Interaction (PPI) Network and Hub Gene Identification and Validation

PPI enrichment analysis was carried out using the following databases: BioGRID [24], InWeb_IM [25], and OmniPath [26] in Metascape (http://metascape.org/). Molecular Complex Detection [27] (MCODE) was used to screen the densely connected network with the default parameters in the whole PPI network. For each MCODE component, pathway and process enrichment analysis has been applied. The three functional description terms with best-scoring *p* value have been retained. The genes within each component were selected as hub genes. To further confirm the hub genes we discovered, the expression levels of hub genes were verified in the validation dataset, GSE90051.

### 2.6. The Construction of the miRNA-Hub Gene Network

The interactions of miRNA-hub genes were predicted using the miRNet [28] web-based platform. By uploading the list of gene IDs of interest, users can map genes to their miRNAs according to the miRTarBase v8.0 [29], TarBase v8.0 [30], and miRecords [31]. The results were presented as each row representing the interaction between one miRNA and its target and visualized in Cytoscape 3.7.2 software [32]. The interactions between two genes were acquired from the STRING database [33]. We implemented the yFiles Layout Algorithms app (“https://www.yworks.com/products/yfiles-layout-algorithms-for-cytoscape”) to construct a circular layout. In the network, a node represents a gene or a miRNA; the undirected link between two nodes is an edge.

## 3. Results

### 3.1. Identification of DEGs and Function Enrichment Analysis

**Figure 1:**
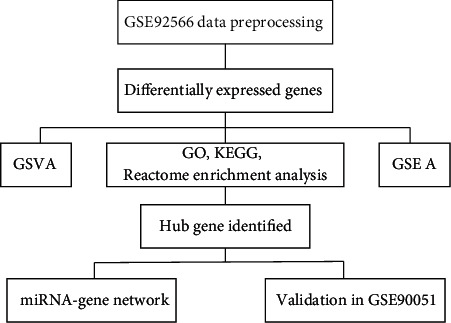
Flow chart of the analysis process of the present study. GO: Gene Ontology; KEGG: Kyoto Encyclopedia of Genes and Genomes; GSVA: gene set variation analysis; GSEA: gene set enrichment analysis.

**Figure 2:**
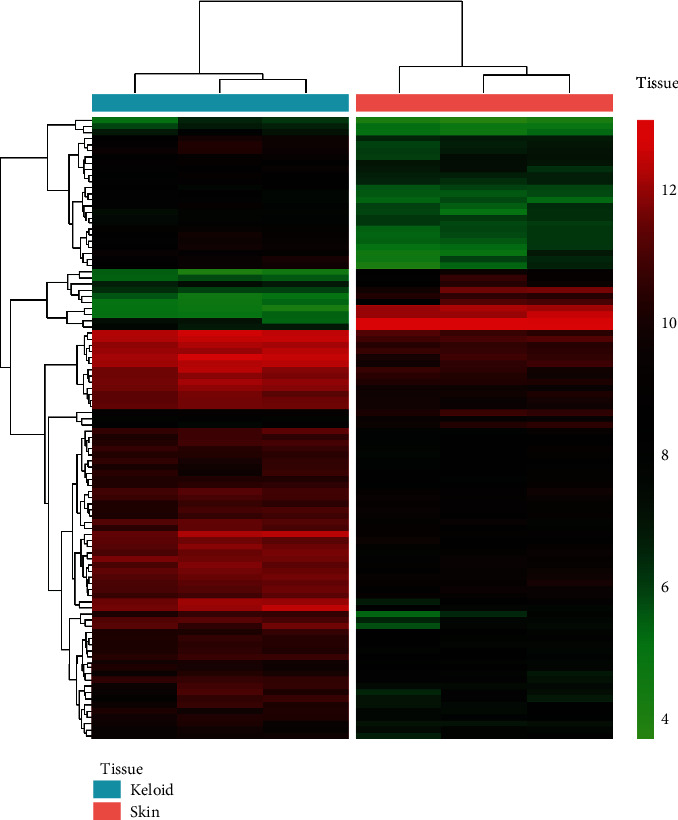
Heatmap for expression profile of differentially expressed genes between the keloid and skin in GSE92566.

**Figure 3:**
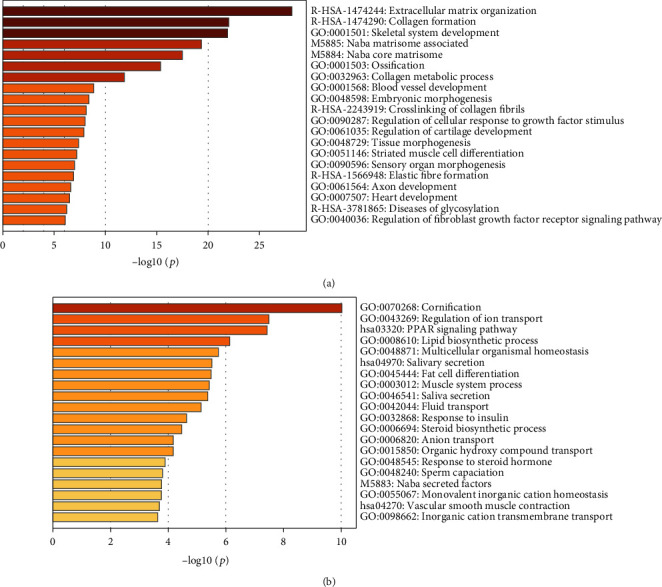
The top 20 functional and pathway enrichment results for a) upregulated and b) downregulated differentially expressed genes between the keloids and skin.

The analysis workflow has been outlined in Figure 1. After downloading and normalizing the GSE92566 and GSE90051, we identified the DEGs between keloid and normal skin in GSE92566 (Figure 2). 117 downregulated DEGs and 204 upregulated DEGs were identified. For upregulated and downregulated DEGs, the GO enrichment analysis, enriched pathways in KEGG, and Reactome database results have been highlighted. As shown in Figure 3, the upregulated DEGs seem to be involved in the extracellular matrix and structure organization, collagen formation, skeletal system, and bone development in BP and function primarily in extracellular constituent and binding processes in MF, while the downregulated DEGs participated in cornification, regulation of ion transport, and PPAR signaling pathway.

### 3.2. GSEA and GSVA for DEGs

**Figure 4:**
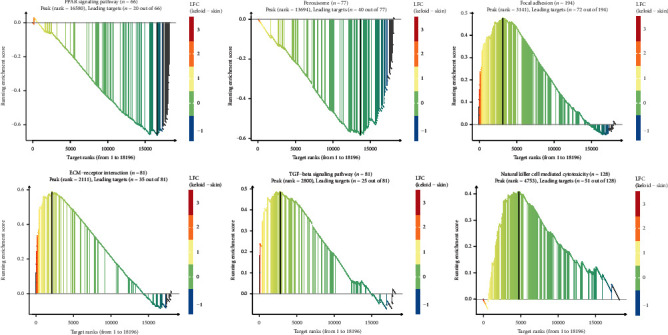
GSEA of expression data from GSE92566 in keloid, as compared to normal skin. The Y-axis is the enrichment score of each gene. The X-axis represents the order of the gene in the dataset. GSEA: gene set enrichment analysis.

**Figure 5:**
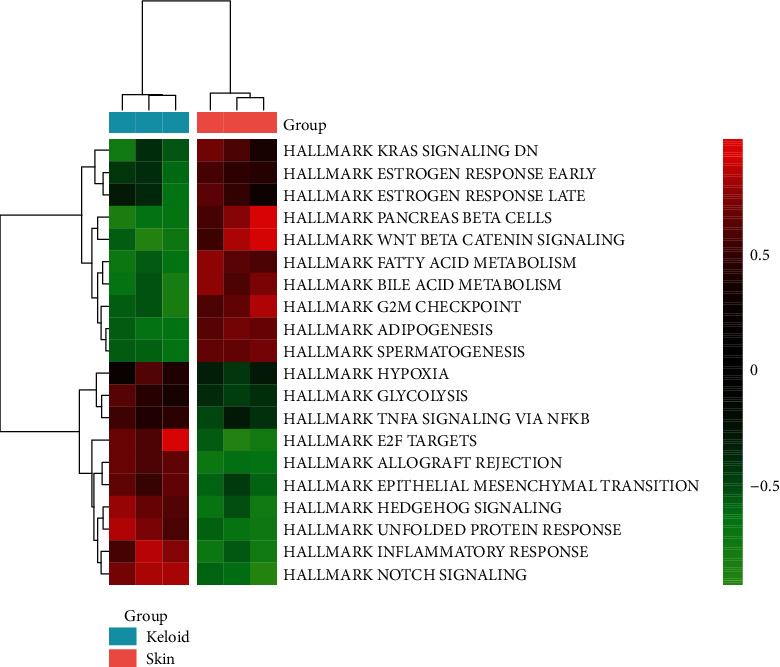
Heatmap of GSVA result. GSVA: gene set variation analysis.

The GSEA and GSVA were performed to explore the potential mechanisms by which the DEGs could be involved in keloid pathogenesis (Figures 4 and 5). We have reported the first six enriched gene sets in the GSEA results sorted by the *p* value (Figure 4). Downregulated DEGs were enriched in the peroxisome and proliferator-activated receptor (PPAR) signaling pathway, while upregulated ones were enriched in natural killer cell-mediated cytotoxicity, TGF-*β* signaling pathway, ECM-receptor interaction, and focal adhesion. Meanwhile, the GSVA results presented 10 downregulated gene sets in keloid tissue compared with normal skin, e.g., xenobiotic metabolism, UV response up, KRAS signaling up, and adipogenesis. Upregulated pathways included TNFA signaling via NFKB, the notch signaling, and inflammatory response (Figure 5).

### 3.3. PPI Network, Hub Gene Identification, and Validation in GSE90051

**Figure 6:**
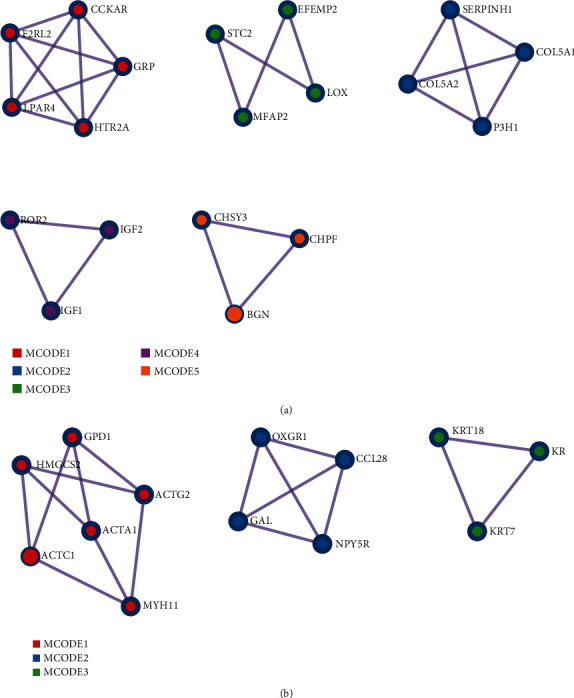
PPI network analysis of (a) upregulated and (b) downregulated differentially expressed genes and the modules identified by MCODE. PPI: protein-protein interaction; MCODE: molecular complex detection.

**Figure 7:**
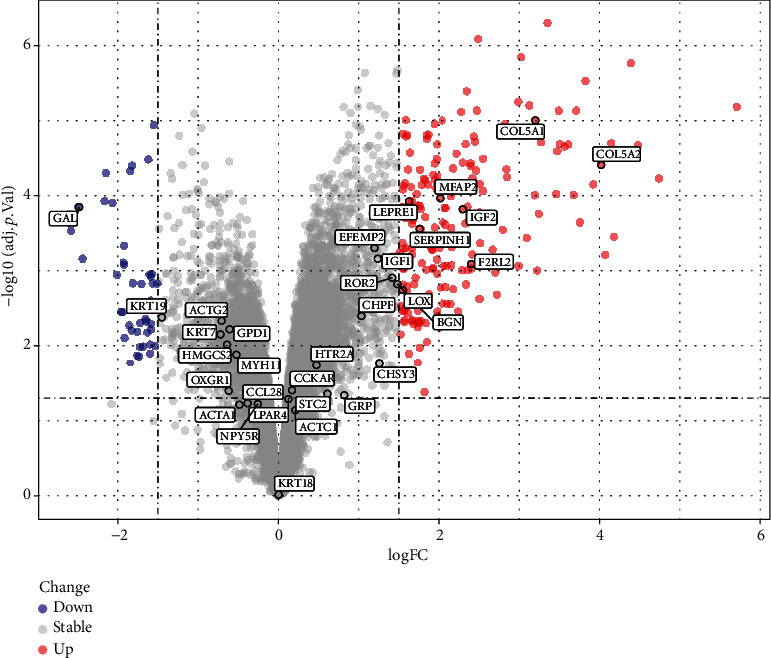
Volcano plots depicting the differentially expressed genes in GSE90051. The red nodes represent upregulated genes. The blue nodes represent downregulated genes.

After downloading the PPI results, the MCODE-identified hub genes are illustrated in Figure 6, and the underlying functions are explained in Table 1. We found 32 hub genes in GSE92566 and 9 of which were detected differentially expressed in GSE90051 (Figure 7). Notably, the collagen-related genes (COL5A1, COL5A2, LEPRE1, and SERPINH1) and elastic fiber-formation-related genes (LOX, STC2, EFEMP2, and MFAP2) were highlighted as upregulated in the network, while keratinization-related genes (KI, KRT7, and KRT18) were downregulated.

### 3.4. The Construction of the miRNA-Hub Gene Network

**Figure 8:**
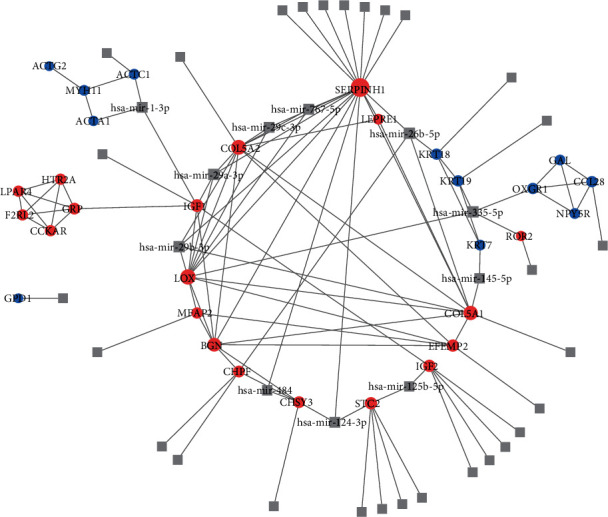
The network of differentially expressed genes and miRNA interaction. The upregulated differentially expressed genes were colored in red and downregulated in blue. The miRNAs were grey squares.

The interactions between the miRNAs and genes were verified at least in two databases. As shown in Figure 8, the upregulated hub genes are illustrated as red circles while downregulated ones were outlined as blue circles. SERPINH1, COL5A1, COL5A2, LOX, and BGN were the top 5 most connected hub genes in the network, in which the collagen-related hub genes and elastic fiber-formation-related hub genes play a central role. Meanwhile, hsa-miR-29a-3p, hsa-miR-29b-3p, hsa-miR-29c-3p, hsa-miR-767-5p, and hsa-miR-484, all associated with both collagen-related and elastic fiber-formation-related hub genes, emerged as the most connected miRNAs.

## 4. Discussion

Our study detected 117 downregulated DEGs and 204 upregulated DEGs between normal skin tissue and keloids and explored their related biological functions and pathways. We also built the network of the miRNA-hub genes, with the expression of hub genes validated. This research provides insights into the underlying pathophysiology of keloid.

The abnormal deposition of collagen within the wound is a characteristic feature of keloids [34]. In our study, the upregulated hub genes were found to be highly centered around collagen-related genes, including serpin peptidase inhibitor clade H, member 1 (SERPINH1); collagen, type V, alpha 1 (COL5A1); collagen, type V, alpha 2 (COL5A2); and prolyl 3-hydroxylase 1 (LEPRE1). SERPINH1 codes for Hsp47, an essential molecular chaperone located in the endoplasmic reticulum for the maturation of collagen, especially type 1 collagen [35]. Previous studies have shown the relationship between the overexpression of SERPINH1 and many kinds of cancers [36–38]. They have also indicated that SERPINH1 takes an important part in collagen-associated diseases, such as osteogenesis imperfecta [39] and vocal fold mucosal fibrosis [40]. Importantly, Kishimoto et al. used siRNA against SERPINH1 in vitro and rat models with vocal fold mucosal fibrosis, resulting in reduced collagen formation in both naive and scar vocal fold fibroblast and reversing the collagen formation in fibrotic mucosa [40]. Similarly, we detected the upregulation of SERPINH1 in our study. This reflected possible similar pathogenesis between keloids and vocal fold mucosal fibrosis with the participation of SERPINH1.

Interestingly, in the earlier studies, SERPINH1 is also relative to the hsa-miR-29 miRNA family [41, 42]. In our miRNA-hub gene network, hsa-miR-29a-3p, hsa-miR-29b-3p, and hsa-miR-29c-3p also play a central role in connecting most of the upregulated hub genes. The hsa-miR-29 family, also called the antitumor miR-29 family, is associated with the regulation of fibrinogen. A study conducted by Fort et al. indicated that the overexpression of the hsa-miR-29 family in transfected cells can decrease the transcript levels of all 5 fibrinogens [43]. Meanwhile, another study reported lower levels of the miR-29 family in keloid compared with normal fibroblasts, which lead to increased expression of collagen [44]. Our study shows a similar relationship between the miR-29 family and keloid and provides convincing evidence for potential correlative therapies [45].

Shih and Bayat gave a comprehensive summary of whole-genome microarray analysis regarding keloid fibroblasts or tissues [9]. They determined that only 25 common dysregulated genes were in keloids in 7 studies where collagen, type I, alpha 1 (COL1A1); collagen, type I, alpha 2 (COL1A2); collagen, type V, alpha 2 (COL5A2); and collagen, type VI, alpha 1 (COL6A1) were systematically upregulated. Similarly, in our study, the upregulated hub genes also highly centered around COL5A1 and COL5A2. It has been proved that the mutation of COL5A1 and COL5A2 leads to Ehlers-Danlos syndrome, a connective tissue disorder [46, 47]. Our study provides a novel mechanism insight whereby the dysregulation of COL5A1 and COL5A2 might be involved in the keloid pathogenesis.

According to the GO and GSEA results, the pathways related to peroxisome and PPAR were enriched with downregulated DEGs. With troglitazone, a widely used PPAR-*γ* agonist, Zhu et al. demonstrated its inhibitory role in collagen synthesis in human keloid fibroblasts by suppressing the effect on TGF-*β*1 signaling through the upregulation of miR-92b [48, 49]. It is believed that fibroblasts play a central part in keloid generation through collagen formation and deposition, ECM synthesis [50], and TGF-*β* signaling pathway [51, 52]. The latter two gene sets were enriched in our results as well. The restoration of these downregulated pathways by tuning the other key pathways such as PPAR could provide possible therapeutic effects to keloids.

The present study has some limitations. Firstly, the comparatively small number of samples from two datasets and limited transcriptome data in a time-series pattern might undermine the results of our study. Secondly, our results are based on pure public data with unavoidable biases, such as age and gender differences. Additionally, further in vivo and in vitro experimental exploration and validation are required. Moreover, transcriptome analysis for keloid, using microarray or not, has identified differentially expressed micro-RNAs [53] during keloid formation and long noncoding RNAs (lncRNAs) [54] between earlobe keloid and normal tissue. DEGs in fibroblast and keratinocytes between keloid and normal were also identified [55, 56]. With an increase in the amount of data, a more systematic bioinformatic analysis could provide insights into the pathophysiology of keloids.

## 5. Conclusions

In conclusion, with the analysis of gene expression data from GSE92566 and GSE90051, DEGs between keloids and normal skin tissue were identified and functionally annotated. The biological pathways enriched in the DEGs and related miRNAs were illustrated which provided potential therapeutic choices for keloid treatment.

## Figures and Tables

**Table 1:** Enriched terms for upregulated and downregulated networks detected by MCODE.

Enriched terms for upregulated network detected by MCODE						
MCODE_1	GO:0007200	Phospholipase C-activatingG protein-coupled receptor signaling pathway	R-HSA-416476	G alpha (q) signaling events	R-HSA-373076	Class A/1 (rhodopsin-like receptors)
MCODE_2	R-HSA-1650814	Collagen biosynthesis and modifying enzymes	R-HSA-1474290	Collagen formation	R-HSA-1474244	Extracellular matrix organization
MCODE_3	R-HSA-1566948	Elastic fiber formation	R-HSA-1474244	Extracellular matrix organization	GO:0030198	Extracellular matrix organization
MCODE_4	GO:0071902	Positive regulation of protein serine/threonine kinase activity	GO:0018108	Peptidyl-tyrosine phosphorylation	GO:0018212	Peptidyl-tyrosine modification
MCODE_5	R-HSA-2022870	Chondroitin sulfate biosynthesis	GO:0030206	Chondroitin sulfate biosynthetic process	GO:0050650	Chondroitin sulfate proteoglycan biosynthetic process
Enriched terms for downregulated network detected by MCODE						
MCODE_1	GO:0090131	Mesenchyme migration	GO:0014866	Skeletal myofibril assembly	R-HSA-397014	Muscle contraction
MCODE_2	R-HSA-373076	Class A/1 (rhodopsin-like receptors)	R-HSA-418594	G alpha (i) signaling events	R-HSA-500792	GPCR ligand binding
MCODE_3	GO:0070268	Cornification	R-HSA-6809371	Formation of the cornified envelope	R-HSA-6805567	Keratinization

## Data Availability

The data that support the findings of this study are available from the corresponding author upon reasonable request.
